# Low temperature and temperature decline increase acute aortic dissection risk and burden: A nationwide case crossover analysis at hourly level among 40,270 patients

**DOI:** 10.1016/j.lanwpc.2022.100562

**Published:** 2022-08-10

**Authors:** Qingli Zhang, Li Peng, Jialu Hu, Huichu Li, Yixuan Jiang, Weiyi Fang, Hongbing Yan, Jiyan Chen, Weimin Wang, Dingcheng Xiang, Xi Su, Bo Yu, Yan Wang, Yawei Xu, Lefeng Wang, Chunjie Li, Yundai Chen, Dong Zhao, Wenzhen Ge, Michelle L. Bell, Antonio Gasparrini, Junbo Ge, Yong Huo, Haidong Kan

**Affiliations:** a School of Public Health, Key Lab of Public Health Safety of the Ministry of Education and NHC Key Lab of Health Technology Assessment, Fudan University, Shanghai, China; b Shanghai Key Laboratory of Meteorology and Health, Shanghai Meteorological Bureau, Shanghai, China; c Department of Cardiology, Zhongshan Hospital, Fudan University, Shanghai Institute of Cardiovascular Diseases, Shanghai, China; d Department of Environmental Health, Harvard T.H. Chan School of Public Health, Boston, MA, USA; e Department of Cardiology, Huadong Hospital Affiliated to Fudan University, Shanghai, China; f Department of Cardiology, Fuwai Hospital Chinese Academy of Medical Sciences, Shenzhen, Shenzhen, China; g Department of Cardiology, Guangdong Provincial People's Hospital, Guangzhou, China; h Department of Cardiology, Peking University People's Hospital, Beijing, China; i Department of Cardiology, General Hospital of the PLA Southern Theater Command, Guangzhou, China; j Department of Cardiology, Wuhan ASIA Heart Hospital, Wuhan, China; k Department of Cardiology, 2nd Affiliated Hospital of Harbin Medical University, Harbin, China; l Department of Cardiology, Xiamen Cardiovascular Hospital Xiamen University, Xiamen, China; m Department of Cardiology, Shanghai Tenth People's Hospital, Shanghai, China; n Department of Cardiology, Beijing Chaoyang Hospital, Capital Medical University, Beijing, China; o Department of Emergency, Tianjin Chest Hospital, Tianjin, China; p Department of Cardiology, Chinese PLA General Hospital, Beijing, China; q Department of Epidemiology, Beijing An Zhen Hospital, Capital Medical University, Beijing Institute of Heart, Lung and Blood Vessel Diseases, Beijing, China; r Regeneron Pharmaceuticals Inc., New York, 10591, USA; s School of Forestry and Environmental Studies, Yale University, New Haven, CT, USA; t Department of Public Health Environments and Society, London School of Hygiene & Tropical Medicine, London, UK; u Centre for Statistical Methodology, London School of Hygiene & Tropical Medicine, London, UK; v Centre on Climate Change and Planetary Health, London School of Hygiene & Tropical Medicine, London, UK; w Department of Cardiology, Peking University First Hospital, Beijing, China; x Children's Hospital of Fudan University, National Center for Children's Health, Shanghai, China

**Keywords:** Non-optimal temperature, Temperature variations, Acute aortic dissection onset, Disease burden

## Abstract

**Background:**

Acute aortic dissection (AAD) is a life-threatening cardiovascular emergency with high mortality, so identifying modifiable risk factors of AAD is of great public health significance. The associations of non-optimal temperature and temperature variability with AAD onset and the disease burden have not been fully understood.

**Methods:**

We conducted a time-stratified case-crossover study using a nationwide registry dataset from 1,868 hospitals in 313 Chinese cities. Conditional logistic regression and distributed lag models were used to investigate associations of temperature and temperature changes between neighboring days (TCN) with the hourly AAD onset and calculate the attributable fractions. We also evaluated the heterogeneity of the associations.

**Findings:**

A total of 40,270 eligible AAD cases were included. The exposure-response curves for temperature and TCN with AAD onset risk were both inverse and approximately linear. The risks were present on the concurrent hour (for temperature) or day (for TCN) and lasted for almost 1 day. The cumulative relative risks of AAD were 1.027 and 1.026 per 1°C lower temperature and temperature decline between neighboring days, respectively. The associations were significant during the non-heating period, but were not present during the heating period in cities with central heating. 23.13% of AAD cases nationwide were attributable to low temperature and 1.58% were attributable to temperature decline from the previous day.

**Interpretation:**

This is the largest nationwide study demonstrating robust associations of low temperature and temperature decline with AAD onset. We, for the first time, calculated the corresponding disease burden and further showed that central heating may be a modifier for temperature-related AAD risk and burden.

**Funding:**

This work was supported by the National Natural Science Foundation of China (92043301 and 92143301), Shanghai International Science and Technology Partnership Project (No. 21230780200), the Medical Research Council-UK (MR/R013349/1), and the Natural Environment Research Council UK (NE/R009384/1).

**Research in context**

**Evidence before this study**

Acute aortic dissection (AAD) is a life-threatening cardiovascular emergency with high mortality. Previous studies have observed that AAD incidence was higher in winter than in summer, and presumably ambient temperature may explain such seasonal variation. We searched PubMed with the terms “aortic dissection” and (“temperature” or “temperature variability” or “meteorological factor”) for all reports published before Dec 31, 2021. Our literature review shows that several studies have reported the association of AAD with temperature and temperature variability, but these studies are generally limited by small sample size and the disease burden of AAD attributable to temperature and temperature variability have never been assessed. Moreover, all previous studies lack the sub-daily information of AAD onset and therefore only examined the associations on a daily basis. In addition, no study has investigated whether indoor heating (a primary adaptive procedure for low temperature) could alleviate the potential harmful effects of low temperature on AAD.

**Added value of this study**

We conducted a time-stratified case-crossover study using a nationwide registry dataset from 1,868 hospitals in 313 Chinese cities to examine the associations of non-optimal temperature and temperature variability with AAD onset and the disease burden. A total of 40,270 eligible AAD cases were included. We found the exposure-response curves for temperature and temperature changes between neighboring days (TCN) with AAD onset risk were both inverse and approximately linear. The risks were present on the concurrent hour (for temperature) or day (for TCN) and lasted for almost 1 day. The associations were significant during the non-heating season, but were not present during the heating season in cities with central heating. 23.13% of AAD cases nationwide were attributable to low temperature and 1.58% were attributable to temperature decline from the previous day.

**Implications of all the available evidence**

The results of this study, along with earlier work, support the hypothesis that low temperature and temperature decline increase the risk of AAD onset. Our studies calculated the corresponding disease burden for the first time and further showed that central heating may be an important modifier for temperature-related AAD risk. These findings suggested that more medical resources should be allocated to AAD rescue at cold days. Indoor heating might be a feasible mitigation to reduce AAD risk and burden.

Alt-text: Unlabelled box

## Introduction

Acute aortic dissection (AAD) is a life-threatening cardiovascular emergency caused by a tear in aortic intima,^1^ with an inpatient mortality ranging from 14 to 22%.^2^^,^^3^ Therefore, identifying modifiable risk factors of AAD is of great public health significance. Previous studies have observed that AAD incidence was higher in winter than in summer, and presumably ambient temperature may explain such seasonal variation.^4^, ^5^, ^6^ However, very few studies have examined the association of AAD with temperature and temperature variations, and these studies were generally limited by small sample size.^6^, ^7^, ^8^, ^9^ Moreover, all previous studies lack the sub-daily information of AAD onset and therefore only examined the associations on a daily basis, which might attenuate the causality due to the unclear chronological order of exposure and events within the same day.^6^^,^^9^^,^^10^ As the onset of AAD fluctuates greatly during 24-hours throughout a day, it is therefore critically important to evaluate the association between temperature and AAD onset at hourly level. In addition, AAD can be classified into type A (which occurs in the ascending aorta) and type B (which occurs in the descending aorta and distal arteries) based on the Stanford classification, which varied in symptoms, anatomical location, and fatality rate and thus might have different risk factors. However, most previous studies did not distinguish the potentially different associations between ambient temperature and different types of AAD.^8^^,^^9^^,^^11^

Quantification of disease burden is critically important for communicating the risk to the public and developing appropriate intervention procedures. To the best of our knowledge, no studies have assessed the disease burden of AAD attributed to non-optimal temperature and temperature variability. In human history, indoor heating has long been used as a primary adaptive procedure for low temperature and thus might be an important intervention for AAD risks attributable to low temperature. Nevertheless, no epidemiological studies had evaluated whether indoor heating could alleviate the harmful effects of low temperature on AAD.

In this study, using a nationwide registry database in China, we aimed to investigate the exposure-response relationships of ambient temperature and temperature change in neighboring days (TCN) with AAD onset at hourly level. We also sought to estimate fractions of AAD cases attributable to non-optimal temperature and temperature variations, and further to explore the effect heterogeneity by several potential modifiers.

## Methods

### Study population

We obtained data of AAD onset from the Chinese Cardiovascular Association (CCA) Database-Chest Pain Center, a national representative registry which started to establish in 2015.^12^ The Chest Pain Center is an emergency department in hospitals that provides urgent healthcare for patients with acute chest pain. All Chinese hospitals with chest pain centers are required to report demographic characteristics (e.g., sex and age), date and time of symptom onset, diagnosis, and measures of clinical examinations and treatment procedures of all patients with chest pain to the registry.^13^ The CCA Executive Committee routinely certifies the chest pain centers based on the data quality. Till the start of this analysis, a total of 1,927 hospitals with a chest pain center were gradually certified by CCA. We included all AAD events from January 2015 to September 2020 that were reported by the certified hospitals. Patients who were referred from other hospitals after initial onset were excluded. Diagnosis of AAD was ascertained by cardiologists based on symptoms, laboratory tests, or imaging findings of computed tomography angiography or echocardiography according to clinical guidelines.^14^^,^^15^ Patients were further classified into type A and type B based on the Stanford classification.^16^ Intramural hematoma was not diagnosed as acute aortic dissection in this registry. We defined the index time for AAD in this analysis as the self-reported time of symptom onset, which was available with hourly precision. Data authorization was obtained by the CCA database before the initiation of this analysis and thus no ethics consideration or informed consent was needed. The study protocol was approved by the Institutional Review Board at the School of Public Health, Fudan University (IRB#2021-04-0889) with a waiver of informed consent. All data in this study were de-identified to protect patient privacy.

### Study design

We used a time-stratified case-crossover design to investigate the associations of temperature and temperature variability with AAD. This study design allows each case to serve as their own control, so that individual factors that are time-invariant within a short period of time, such as disease history, age, and sex, are automatically controlled for.^17^^,^^18^ For each AAD case, we selected 3 to 4 controls that were before and after the index time and were in the same calendar month, year, day of week, and time of day as the index time to control for potential time trends and seasonality.^18^

### Environmental data

Meteorological Data including hourly temperature and relative humidity during the study period were obtained from monitoring stations in the China Meteorological Data Sharing Service System (http://data.cma.cn/). Hourly air pollutants concentrations (PM_2.5_, particulate matter with an aerodynamic diameter <2.5 mm; NO_2_, nitrogen dioxide, ozone; CO, carbon monoxide; SO_2_, sulfur dioxide) were obtained from China's National Urban Air Quality Real-time Publishing Platform. All hourly exposure data were then linked to the hospital address for each AAD case and its controls from the nearest meteorological monitoring station and air pollutant station. To reduce exposure measurement error, we excluded hospitals located more than 100 km from the nearest meteorological monitoring station. TCN was calculated by the difference of mean temperature between the previous 24-hour and the current 24-hour (the starting point is defined as the onset hour of AAD). Therefore, a positive TCN represented a temperature increase and a negative TCN represented a temperature decline from the previous day to the current day.

### Statistical analysis

#### Risk estimation

Conditional logistic regression and distributed lag models (DLM) were used to investigate exposure-response relationships and the lagged effects of temperature and TCN on AAD. We first used a DLM model to build a cross-basis function for the exposure and the outcome across different lags. Based on a pilot study, we observed approximately linear exposure response curves for the associations of temperature and TCN with AAD (details were presented in online eFig 1). We thus used a simple linear function to capture the exposure-response relationships. For lagged effects, we used natural cubic splines with 3 degrees of freedom (*df*) placing knots at equal intervals in the log scale of lags for temperature and TCN. We used hourly lags from the time of AAD onset (lag 0 h) to 72 hours previously (lag 72 h) for temperature and daily lags from the day of onset (lag 0 day, the starting point is defined as the onset hour of AAD) to 3 days previously (lag 3 day) for TCN. We then added these cross-basis functions to conditional logistic regression models adjusting for relative humidity (3-day moving average in a natural cubic spline of 3 *df*) and a binary variable of public holidays. Temperature and TCN were also mutually controlled for in the corresponding models. The cumulative relative risks (RRs) and 95% confidence intervals (95%CI) of AAD per 1°C change of hourly temperature and daily mean temperature from the previous day were calculated. In addition, we plotted the exposure-response curves using distributed lag non-linear models (DLNM) to capture the overall shape of the association between exposures and AAD. We used natural cubic splines with 5 *df* placing knots at equal spaces in temperature and TCN range to capture possible non-linear relationships. Other parameters for cross-basis functions and covariates in DLNM were the same as those in the DLM.

We further examined possible effect heterogeneity by central heating, seasons, regions, medical quality, AAD subtype (type A and type B by Stanford classification), age (< 60 and ≥ 60 years), and sex. In cities with central heating, we compared the modification effects of central heating by dividing into heating and non-heating periods. The central-heating policy was introduced in China during the 1950s, in which cities north of a line along the Qin Mountains and the Huai River (known as the Qin-Huai line) have centrally controlled public heating system (central heating), while cities south of the line have no central heating. For families with low economic status in cities with central heating, the costs of central heating may be subsidized by the government. Heating period is generally from November to March, with minor adjustments based on specific temperature of each year in each city. Due to the nature of time-stratified case-crossover study, the heating period can only be defined at monthly level, which is rounded according to the actual heating dates of each year in each city. For cities without central heating, season was divided into winter (from December to February in the next year) and other seasons (from March to November). We also did seasonal stratification by dividing into 4 seasons (spring, summer, autumn, and winter). In order to examine regional heterogeneity, we did stratification by dividing into 5 climate zones. In addition, we did stratification analyses by medical quality, which were indicated by hospital grades. Hospital grades were evaluated by the National Hospital Performance Evaluation Project of China based on hospital infrastructure, medical service and management, technical level and efficiency, and quality and safety of clinical care. Tertiary A hospitals were defined as high-ranked hospitals, and other hospitals were defined as low-ranked hospitals in the study. To test the statistical significance of the potential effect heterogeneity between strata, we first used the following formula to calculate the 95% CIs of the effect differences: $\left(Q_{1}-Q_{2}\right)\pm 1.96\;\sqrt{SE_{1}^{2}+SE_{2}^{2}}\;$, with Q_1_ and Q_2_ indicating the estimates and SE_1_ and SE_2_ indicating the corresponding standard errors in each stratum.^19^^,^^20^ We then obtained the P values of the effect differences based on the calculated 95% CIs.^21^

A number of studies indicated that air pollution was positively associated with cardiovascular diseases,^22^ and several studies also reported positive associations between air pollution and AAD.^6^^,^^23^ We thus performed sensitivity analyses by additionally adjusting for air pollutants (PM_2.5_, NO_2_, CO, O_3_, and SO_2_) on the concurrent day (Lag 0 day), both one at a time and all five simultaneously, to account for possible residual confounding. In order to examine the influence of cases with long time intervals from onset to admission, we did a sensitivity analysis by excluding cases who admitted to hospital more than 48 hours after the symptom onset.

#### Attributable fractions calculation

We calculated the fractions of total AAD cases attributable to temperature and TCN to quantify the burden of AAD related to each exposure measure. To obtain attributable fractions (AFs) of AAD for temperature in each day and in each city, we first computed the city-specific RRs of AAD in each day at the daily average temperature (i.e., 24 h cumulative lag) comparing with the minimum onset temperature based on the exposure-response coefficients of temperature (lag 0-24 h) estimated in DLM at the hourly resolution. For TCN, we calculated city-specific RRs comparing the TCN with no temperature change (i.e., 0°C) on the concurrent day (the changes in temperature on lag 0 day from lag 1 day). The number of AAD cases attributable to temperature or TCN in each day and in each city were then calculated in the following Eq (1):

$$n_{it}=\left(\frac{RR_{it}-1}{RR_{it}}\right)*N_{it}$$

where n_it_ is the number of AAD cases attributable to temperature/TCN in city *i* on day *t*; RR_it_ is the relative risk of AAD onset associated with temperature/TCN in city *i* and day *t*; N_it_ is the total AAD cases in city *i* and on day *t*.

To account for possible regional and seasonal heterogeneity in the exposure-response relationships, we used RRs estimated in specific region and season when estimating the city- and day-specific n_it_. We then estimated the total number of AAD cases attributable to temperature/TCN by summing n_it_ across all days during the study period and across all cities. Lastly, the AFs for temperature or TCN were calculated by dividing the total number of AAD cases by the total number of AAD cases attributable to temperature or TCN as calculated above.

All statistical analyses were conducted using R software (version 3.4.2, R Foundation for Statistical Computing) with the ‘dlnm’ package. All statistical tests were two-sided and a p value less than 0.05 was considered statistically significant. The funders had no role in study design, data collection and analysis, decision to publish, or preparation of the manuscript.

## Results

### Descriptive statistics

A total of 1,868 hospitals covering 313 Chinese cities with eligible AAD patients were included in the study from January 2015 to September 2020. Online eFig 2 shows the locations of these hospitals. The mean distance between a hospital and its nearest meteorological monitoring stations was 17.66 (standard deviation [SD] = 13.98) km. During the study period, a total of 5,228,973 patients with acute chest pain were registered in the CCA database, among which 56,751 AAD cases were identified. After excluding 2,556 cases from hospitals without a certified chest pain center and 13,925 cases that were referrals or admitted by a hospital more than 100 km (2 hospitals) from the nearest meteorological station, a total of 40,270 AAD cases were included in the analysis (online eFig 3). The included hospitals were located in all five climate zones (N= 670 in the subtropical monsoon zone; N=655 in the temperate monsoon zone, N=191 in the temperate continental zone, N=345 in the tropical monsoon zone, and N= 7 in alpine zone). 40.14% of cases occurred in cities with central heating, and 64.77% were identified in high-ranked hospitals (tertiary A hospitals). The mean age of eligible AAD patients was 58.97 years (SD=13.80), 74.78% were male. The median time interval from onset to admission for AAD cases was 2.5 h (P5 = 0.3 h, P95 = 64.0 h). As information on Stanford types were optional, but not mandatory to be reported to the national registry, we thus collected only 28,660 cases with Stanford types, among which 13,671 (47.7%) were Type A dissection and 14,989 (52.3%) were Type B dissection. The time distribution of AAD cases was shown in online eFig 4. The highest frequency of incident AAD was present in 7:00 — 12:00 (32.5%), followed by 13:00 — 18:00 (27.9%), 19:00 — 24:00 (23.7%) and, 1:00 — 6:00 (16.0%). For weekly variations, the frequency was slightly higher in Tuesday and Monday, lower in Saturday and Sunday with an overall homogeneous distribution. Monthly distribution shows that the number of AAD cases was highest in December and lowest in August. Both in heating and non-heating areas, the number of AAD patients were highest in winter and autumn, while lowest in summer. The average exposure level of hourly temperature for all AAD cases at the time of onset was 15.86°C, and the hourly relative humidity was 69.16%.

### Regression results

#### Low temperature and TCN with AAD onset

**Figure 1:**
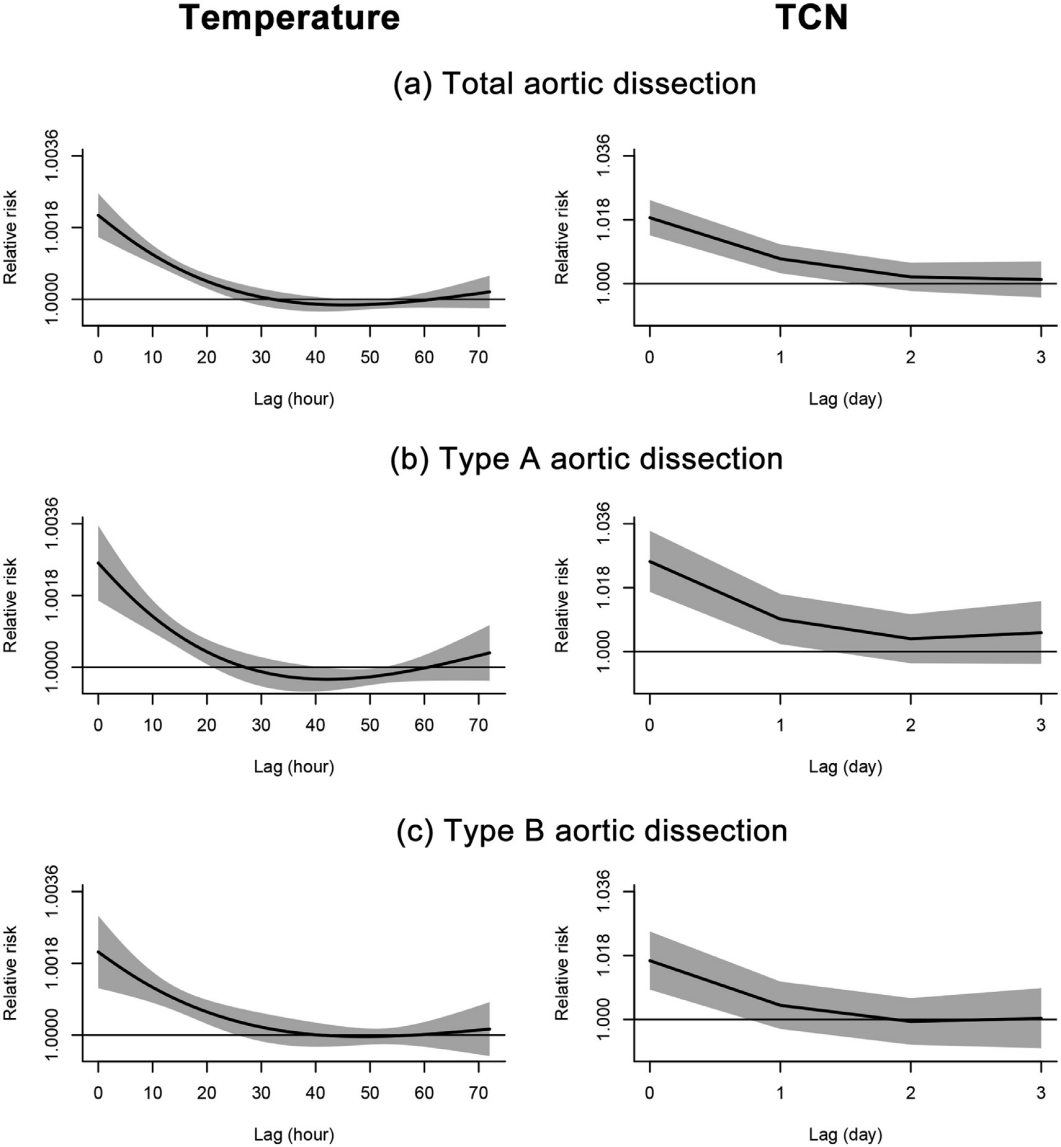
**Lag structure for temperature and TCN with total (a), type A (b), and type B aortic dissection (c) during 2015-2020**. TCN, temperature change in neighboring days. Effects were estimated as relative risk per 1°C lower temperature or temperature decline between neighboring days; Solid lines=relative risk of aortic dissection; shaded areas=95% confidence intervals.

Overall, we found inverse associations between hourly temperature and AAD onset. The harmful effect of low temperature on AAD was estimated to emerge immediately in the same hour, gradually attenuated within the first 24 hours, and became null afterwards (Figure 1). Therefore, we used results with the cumulative lag 0 - 24 hour of temperature as main results. The cumulative RR of AAD was 1.027 (95%CI: 1.021, 1.032) per 1°C decrease in hourly temperature (Table 1). The overall lag patterns and exposure-response coefficients for type A and B AAD were similar to those for total AAD (RR=1.030, 95%CI: 1.020, 1.039 for type A AAD and RR=1.028, 95%CI: 1.019, 1.038 for type B AAD per 1°C decrease in hourly temperature).

**Table 1:** Relative risks of acute aortic dissection associated with ambient temperature (lag 0-24 h) and TCN (lag 0-1 days), 2015-2020.

		Temperature		TCN	
	N	RR (95% CI)	*P*-value^a^	RR (95% CI)	*P*-value^a^
Overall	40,270	1.027 (1.021, 1.032)		1.026 (1.019, 1.033)	
By acute aortic dissection subtype					
Type A	13671	1.030 (1.020, 1.039)	0.842	1.035 (1.022, 1.047)	0.110
Type B	14989	1.028 (1.019, 1.038)		1.021 (1.009, 1.033)	
By age					
>=60	19676	1.023 (1.015, 1.031)	0.176	1.028 (1.018, 1.038)	0.550
<60	20578	1.030 (1.023, 1.038)		1.024 (1.013, 1.034)	
By sex					
Male	30113	1.028 (1.022, 1.035)	0.233	1.022 (1.014, 1.031)	0.109
Female	10155	1.021 (1.010, 1.032)		1.036 (1.022, 1.050)	
By central heating					
Cities with central heating					
Heating periods	5724	1.002 (0.990, 1.014)	0.002	0.997 (0.980, 1.014)	0.007
No-heating periods	10440	1.027 (1.017, 1.036)		1.026 (1.013, 1.039)	
Cities without central heating					
Winter	6703	1.035 (1.020, 1.050)	0.491	1.029 (1.010, 1.048)	0.363
Other seasons	17403	1.041 (1.031, 1.051)		1.039 (1.027, 1.052)	
By seasons					
Winter	10,927	1.021 (1.010, 1.032)	Ref.	1.012 (0.999, 1.026)	Ref.
Spring	11,095	1.031 (1.022, 1.040)	0.156	1.029 (1.018, 1.040)	0.073
Summer	8,547	1.041 (1.026, 1.057)	0.035	1.032 (1.011, 1.053)	0.135
Autumn	9,701	1.022 (1.010, 1.033)	0.913	1.034 (1.017, 1.051)	0.052
By climate zones					
Temperate continental zone	3,976	1.003 (0.991, 1.016)	Ref.	0.995 (0.978, 1.013)	Ref.
Subtropical monsoon zone	14,163	1.043 (1.033, 1.053)	0.000	1.043 (1.030, 1.055)	0.000
Temperate monsoon zone	12,427	1.023 (1.014, 1.032)	0.015	1.027 (1.014, 1.039)	0.005
Tropical monsoon zone	9,640	1.031 (1.016, 1.046)	0.006	1.021 (1.003, 1.040)	0.043
Alpine zone	64	1.046 (0.896, 1.222)	0.612	0.961 (0.787, 1.174)	0.746
By hospital grade^b^					
High-ranked hospital	26,084	1.024 (1.017, 1.031)	0.167	1.022 (1.013, 1.031)	0.152
Low-ranked hospital	14,186	1.032 (1.023, 1.041)		1.033 (1.021, 1.045)	

TCN, temperature change in neighboring days; RR, relative risk; Effects were estimated as relative risk per 1°C lower temperature or temperature decline between neighboring days.

a P-value for the comparison between different strata.

b Hospital grades were evaluated by the National Hospital Performance Evaluation Project. Tertiary A hospitals were defined as high-ranked hospitals, and other hospitals were defined as low-ranked hospitals in the study.

**Table 2:** Fractions of aortic dissection onset attributable to ambient temperature and TCN nationwide, 2015-2020.

Area	AF (95% CI) (%)	
	Temperature	Negative TCN
Nationwide	23.13 (17.17, 28.36)	1.58 (1.00, 2.13)
Cities with central heating		
All year	17.02 (11.63, 21.69)	1.38 (0.71, 2.02)
Heating periods	-	-
Non- heating periods	26.41 (18.04, 33.65)	3.97 (2.05, 5.83)
Cities without central heating		
All year	27.26 (20.92, 32.89)	1.71 (1.20, 2.21)
Winter	22.77 (14.05, 30.39)	-
Other seasons	29.00 (23.57, 33.85)	4.40 (3.10, 5.68)

AF, attributable fraction; TCN, temperature change in neighboring days.

**Figure 2:**
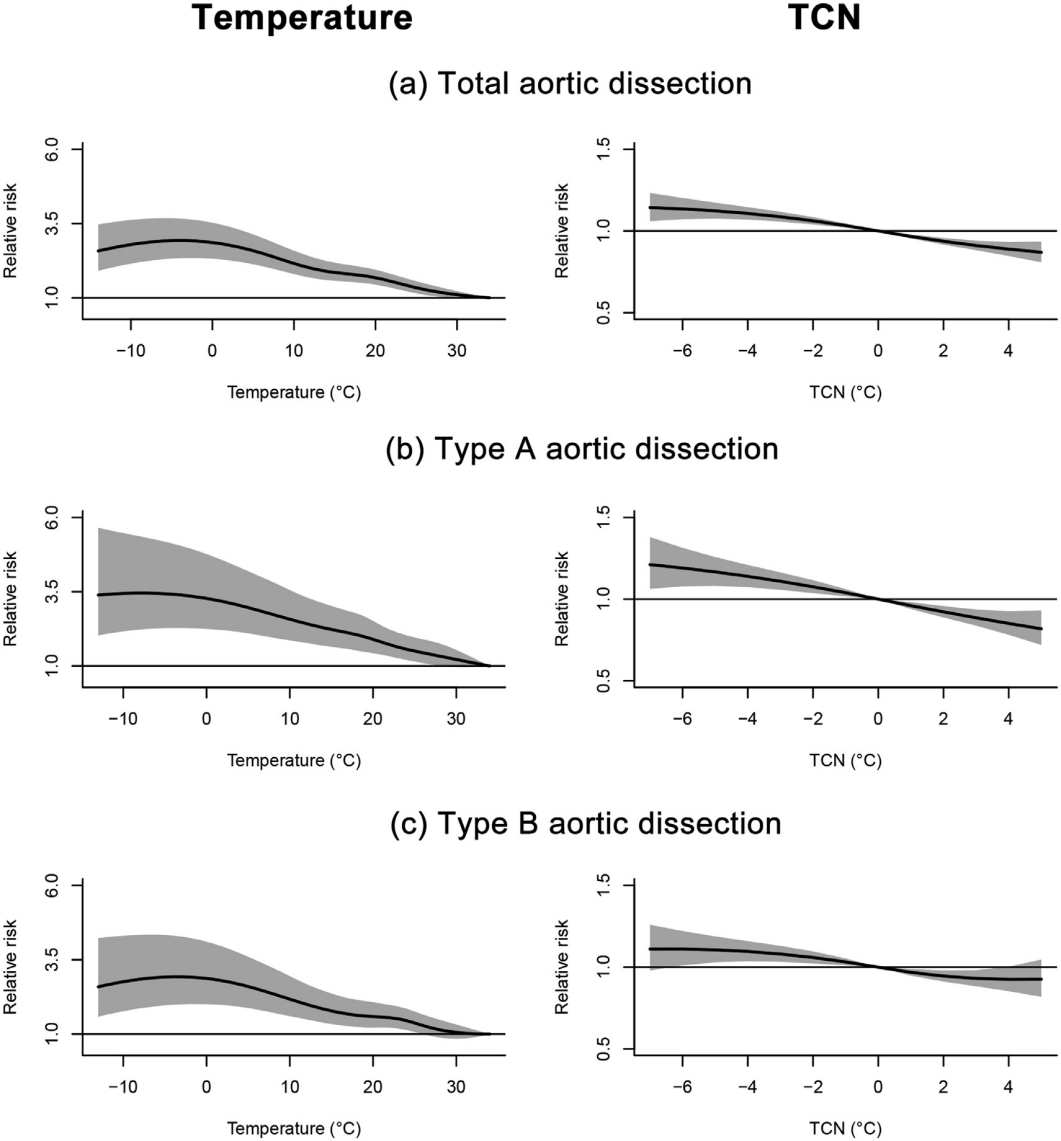
**Exposure-response relationships of temperature (lag 0-24 hours) and TCN (lag 0-1 days) with total (a), type A (b), and type B aortic dissection (c) during 2015-2020**. TCN, temperature change in neighboring days. Solid lines=relative risk of aortic dissection; shaded areas=95% confidence intervals.

The adverse effect of TCN on AAD was the largest on the concurrent day, attenuated in the next day, and disappeared thereafter (Figure 1). Overall, the exposure-response curve for TCN and AAD was almost linear (Figure 2). The cumulative RRs of lag 0 - 1 day of AAD was 1.026 (95%CI: 1.019, 1.033) per 1°C decrease in daily mean temperature from the previous day. The lag patterns and exposure-response coefficients of TCN were comparable between total AAD and the two subtypes. The RRs were 1.035 (95%CI: 1.022, 1.047) for type A and 1.021 (95%CI: 1.009, 1.033) for type B AAD per 1°C decrease in daily mean temperature from the previous day (Table 1).

**Figure 3:**
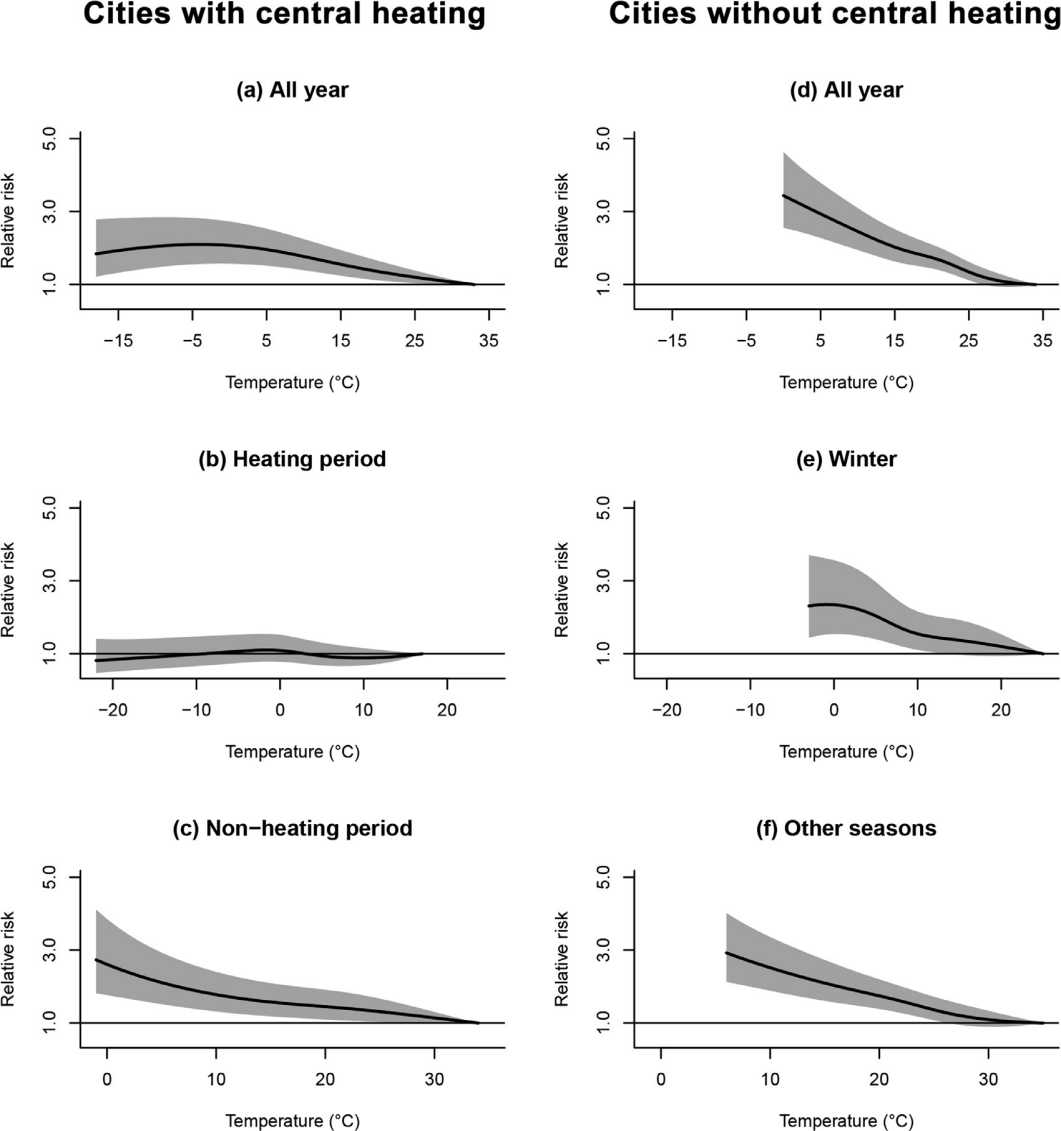
**Exposure-response curves for temperature and acute aortic dissection in lag 0-24 hours during all year (a), heating periods (b), and non-heating periods (c) in cities with central heating, and during all year (d), winter (e), and other seasons (f) in cities without central heating**. Solid lines=relative risk of aortic dissection; shaded areas=95% confidence intervals.

**Figure 4:**
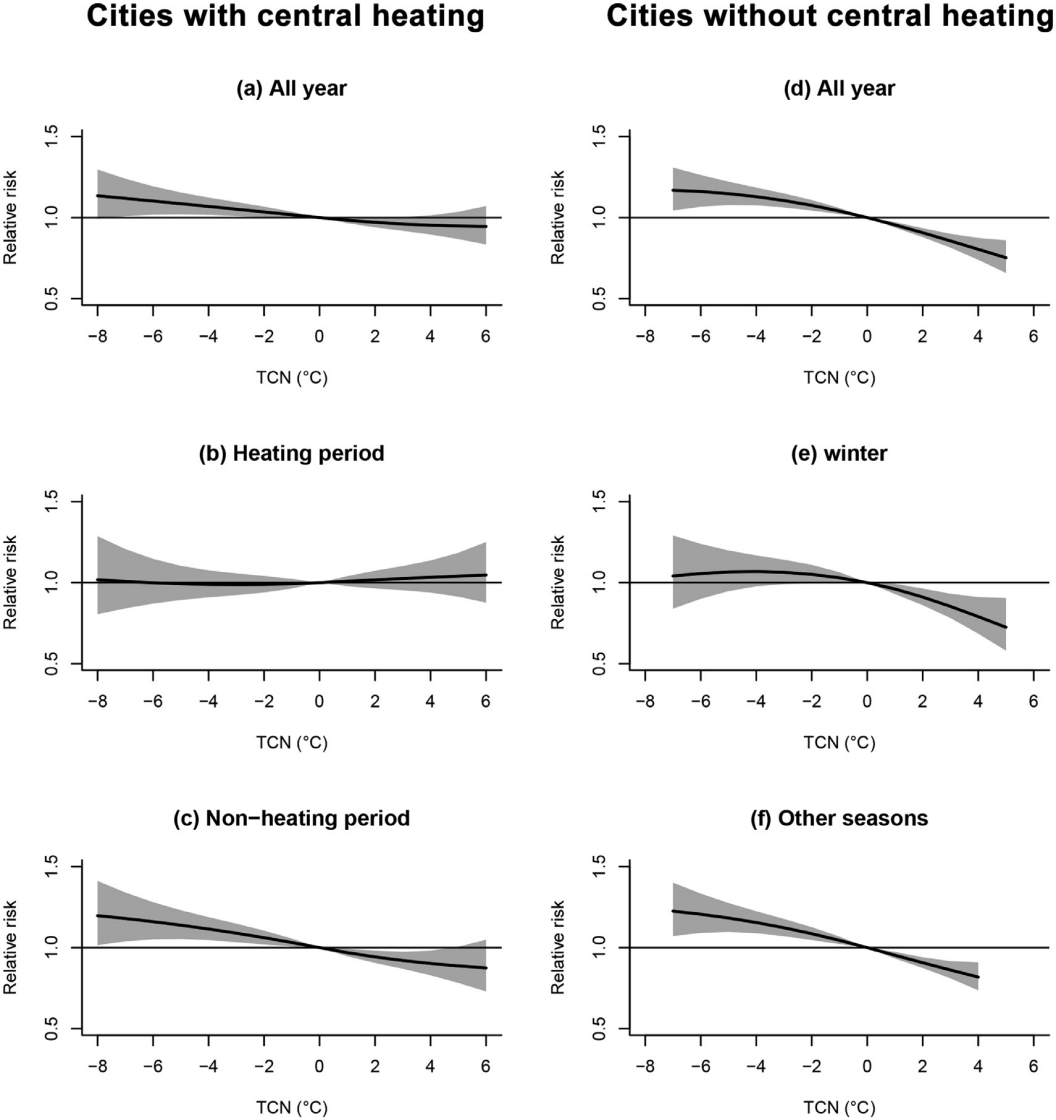
**Exposure-response curves for TCN and acute aortic dissection in lag 0-1 days during all year (a), heating periods (b), and non-heating periods (c) in cities with central heating, and during all year (d), winter (e), and other seasons (f) in cities without central heating**. TCN, temperature change in neighboring days. Solid lines=relative risk of aortic dissection; shaded areas=95% confidence intervals.

Some modification factors were identified. Heterogeneous associations were observed between heating period and non-heating period in cities with central heating (Figures 3, 4, online eFig 5, and online eFig 6). Overall, the exposure-response curves of temperature and TCN with AAD were approximately linear across regions and seasons. As shown on Figure 3, the relative risks of AAD were close to null across all ranges of temperature during heating periods in cities with central heating, while low temperature was associated with elevated AAD onset during non-heating periods in these cities. Similar differential relationships were found for TCN. As shown on Figure 4, in cities with central heating, TCN was not associated with AAD in heating periods, but negative TCN (temperature decline) was associated with increased AAD onset in non-heating periods. The effects of temperature and TCN have no significant difference in winter and other seasons for cities without central heating. When stratified by 4 seasons, the magnitudes for associations of temperature and TCN with AAD were smallest in winter (Table 1, online eFig 7 and eFig 8). We found different exposure-response curves in different climate zones. Significant associations of temperature and TCN with AAD onset were observed in the subtropical monsoon zone, the temperate monsoon zone, and the tropical monsoon zone, but not observed in the temperate continental zone and the alpine zone (Table 1, eFig 9, eFig 10). Stratification analysis by medical quality, age and sex showed no notable effect heterogeneity for either temperature or TCN (Table1 and online eFig 11). In sensitivity analyses, the associations of temperature and TCN with AAD barely changed after additionally adjusting for air pollutants (results not shown). By excluding cases who admitted to hospital more than 48 hours after the symptom onset, and the results were similar to main results (online eFig 12).

### Attributable fractions of AAD by temperature and TCN

The AFs of AAD to temperature was 23.13% (95%CI: 17.17%, 28.36%) nationwide. In cities with and without central heating, the AFs were 17.02% (95%CI: 11.63%, 21.69%) and 27.26% (95%CI: 20.92%, 32.89%) respectively, across all seasons. As for TCN, the AFs of temperature decline (TCN<0) were 1.58% (95%CI: 1.00%, 2.13%) nationwide, 1.38% (95%CI: 0.71%, 2.02%) in cities with central heating, and 1.71% (95%CI: 1.20%, 2.21%) in cities without central heating.

## Discussion

In this large, nationwide case crossover study, we found low temperature and temperature decline between neighboring days were associated with increased onset of AAD. Approximately 23.13% of the AAD cases can be attributed to low temperature and 1.58% can be attributed to temperature rapid decline across all cities. Further, central heating might be a protective measure to reduce the risk of AAD and burden. To our knowledge, this is the largest study to date to investigate the effect of temperature and TCN on AAD onset at an hourly temporal resolution, the first study to quantify AAD burden attributable to non-optimal temperature, and the first study to evaluate the role of central heating in temperature-related AAD onset.

Epidemiological evidence on the associations of temperature and temperature variation with AAD onset is scarce. Previous studies were limited by relatively small sample sizes and results may not be comparable due to differences in exposure and risk window definitions, referent levels, as well as study populations. Nevertheless, the overall trend of the associations between cold weather and AAD onset in our study were consistent with previous ones.^5^^,^^6^^,^^9^^,^^10^^,^^24^ In addition to low ambient temperature, we found temperature decline between two neighboring days was also associated with higher AAD onset. To the best of our knowledge, only one previous study has examined the associations of AAD onset with TCN besides absolute temperature, and the findings were consistent with ours.^9^ Both studies indicated that the relative risk of AAD monotonically increased with lower temperature and temperature decline. There are mild or moderate differences for the shape and slope of the exposure-response curves between two studies, which may reflect the inherent disparity due to different exposure time window, study population, and sample size. Compared with the previous study which only examined the associations on a daily basis in 11 cities (N=8,182),^9^ the current study (N=40,270) covered 313 cities and explored the critical exposure time window with higher time precision (at hourly level). The heterogeneity in climate zones found in the present study indicated that differences in climate zones might also contribute to the different results between the present study and the study by Chen et al. After controlling for air pollution, we obtained robust associations of temperature and TCN with AAD onset, suggesting that the effects of temperature and TCN on AAD incidence were independent of air pollution. In this study, we further quantified the disease burden of AAD attributed to non-optimal temperature (23.13%) and TCN (1.58%), which had never been reported previously. Moreover, we found the potential protective effects of central heating in reducing AAD risks for the first time. The associations of low temperature and temperature decline with increased onset of AAD are biologically plausible. Low temperature and sudden temperature drop can affect the autonomic nervous system and thus result in vasoconstriction, higher blood viscosity, and elevated blood pressure, all of which are established risk factors of AAD.^25^, ^26^, ^27^ In addition, the temperature-related AAD risk might also be explained by the physical activity reduction and diet changes in cold seasons. However, the underlying mechanism warrants further investigation.

Based on the differential associations in the heating and non-heating periods, we speculated that central heating might mitigate the adverse effects of low temperature and temperature decline on AAD. In cities with central heating, low temperature is associated with increased AAD onset in non-heating periods, but the relative risks of AAD are close to null across all temperature ranges in heating period. For TCN, we also found a decrease in daily mean temperature from the previous day was associated with increased AAD onset only in non-heating periods but not in heating periods for cities with central heating. In addition to seasonal differences not related to temperature, one possible explanation can be that central heating may mitigate the harm of low temperature and temperature drop on AAD.

Our findings have important implications for AAD prevention. Using hourly measures of temperature, we found that exposure to low temperature had immediate adverse effect on AAD and that this association persisted for up to 24 hours. Similarly, the associations for TCN were also observed to be relatively short-term (i.e., within lag 0-1 days). These findings indicate the potential public health benefits of developing an effective risk communication system for ambient temperature and temperature change to warn people for cold-adaptation procedures. Our findings suggested that low temperature and temperature drop accounted for 23.13% and 1.58% of AAD, respectively. Moreover, we found the AAD risks associated with temperature and TCN in heating period were significantly lower than that in non-heating period. These findings further support the importance of heating for health benefits during the cold season. Considering air pollution caused by the traditional coal-powered heating, clean energy heating should be encouraged. Future studies are needed to evaluate the appropriate duration and coverage of central heating, taking issues such as cost effectiveness and the overall environmental health impact into full consideration.

This study had several strengths. First, by using the nationwide CCA database, we obtained the largest representative sample worldwide for the relatively uncommon AAD (approximately 2.9/100,000 person /year incidence^28^). Second, detailed information on the timing of AAD onset at the hourly level in the CCA database allowed us to explore the critical exposure time window with higher precision. Moreover, we further provided evidence of AAD disease burdens attributable to low temperature and temperature variability nationwide and in different areas, which, to the best of our knowledge, had not been reported previously. Third, the automatically controlled individual confounders by using the case crossover design, and the clear chronological order of exposure and events by using a finer time window further strengthened the causal inference. Finally, the broad spatial-temporal coverage of this nationwide database provides a great opportunity to explore the possible spatial and seasonal heterogeneity in the temperature-AAD and temperature variability-AAD associations as well as the possible mitigation effect of central heating.

Several limitations should be noted. First, exposure misclassification was inevitable given that we mapped temperature measures from fixed-site monitoring stations to the hospital address for each patient. However, these errors were likely to be non-differential and might bias the results towards the null. Second, the CCA database only registered patients who visited the chest pain centers, while the AAD cases who died outside hospitals and a small proportion of AAD patients without chest pain symptoms were not included in the study. Third, the AAD onset time was self-reported, which might introduce recall bias. As AAD is a very emergency event, and most patients have a typical symptom of the sudden onset of severe chest or back pain. Therefore, we can speculate that patients or their witnesses can easily remember the onset time and the report bias might have very little impact on the results. Fourth, whether to use indoor heating and the heating months were roughly estimated based on the central-heating policy, so misclassification of central heating was possible. Finally, although this is a representative nationwide analysis in China, caution is still needed in generalizing our findings to other countries and regions.

In summary, this nationwide study demonstrated that ambient temperature and temperature decline between neighboring days were important modifiable risk factors that contributed a considerable proportion of AAD onset. The adverse effect of low temperature occurred immediately within the first hour of exposure. Central heating may be an important modifier for temperature-related AAD onset. These findings highlight the importance of establishing prompt alerts of cold temperature for AAD-susceptible populations.

## Contributors

Qingli Zhang: conceptualisation, formal analysis, methodology, software, validation, writing – original draft.

Li Peng: data curation, resources, investigation, formal analysis, writing – original draft.

Jialu Hu: data curation, project administration.

Huichu Li: methodology, writing – review & editing.

Yixuan Jiang: methodology, investigation, formal analysis.

Weiyi Fang: data curation, project administration.

Hongbing Yan: data curation, project administration.

Jiyan Chen: data curation, project administration.

Weimin Wang: data curation, project administration.

Dingcheng Xiang: data curation, project administration.

Xi Su: data curation, project administration.

Bo Yu: data curation, project administration.

Yan Wang: data curation, project administration.

Yawei Xu: data curation, project administration.

Lefeng Wang: data curation, project administration.

Chunjie Li: data curation, project administration.

Yundai Chen: data curation, project administration.

Dong Zhao: data curation, project administration.

Wenzhen Ge: writing – review & editing.

Michelle L. Bell: methodology, writing – review & editing.

Antonio Gasparrini: funding acquisition, methodology, writing – review & editing.

Junbo Ge: conceptualisation, data curation, project administration, supervision, writing – review & editing.

Yong Huo: conceptualisation, data curation, project administration, supervision, writing – review & editing.

Haidong Kan: conceptualisation, funding acquisition, resources, supervision, writing – review & editing.

## Data sharing statement

The data required for the analysis in this study were obtained from the Data Management Committee of the CCA Database - Chest Pain Center. The study is carried out under privacy protection. Anonymized health data will be available through a formal application process which will be reviewed by the Data Management Committee of the CCA Database - Chest Pain Center.

## Declaration of interests

We declare no competing interests.

## Appendix Supplementary materials

Image, application 1
