# Supplementary material for: Low temperature and temperature decline increase acute aortic dissection risk and burden: A nationwide case crossover analysis at hourly level among 40,270 patients
Source: Lancet Reg Health West Pac. 2022 Aug 10;28:100562. doi: 10.1016/j.lanwpc.2022.100562 (PMC9386641; doi:10.1016/j.lanwpc.2022.100562)
Supplement: Supplementary file 1 [file mmc1.docx]

**Supplemental Material for:**

**Low temperature and temperature decline increase acute aortic dissection risk and burden: a nationwide case crossover analysis at hourly level among 40,270 patients**
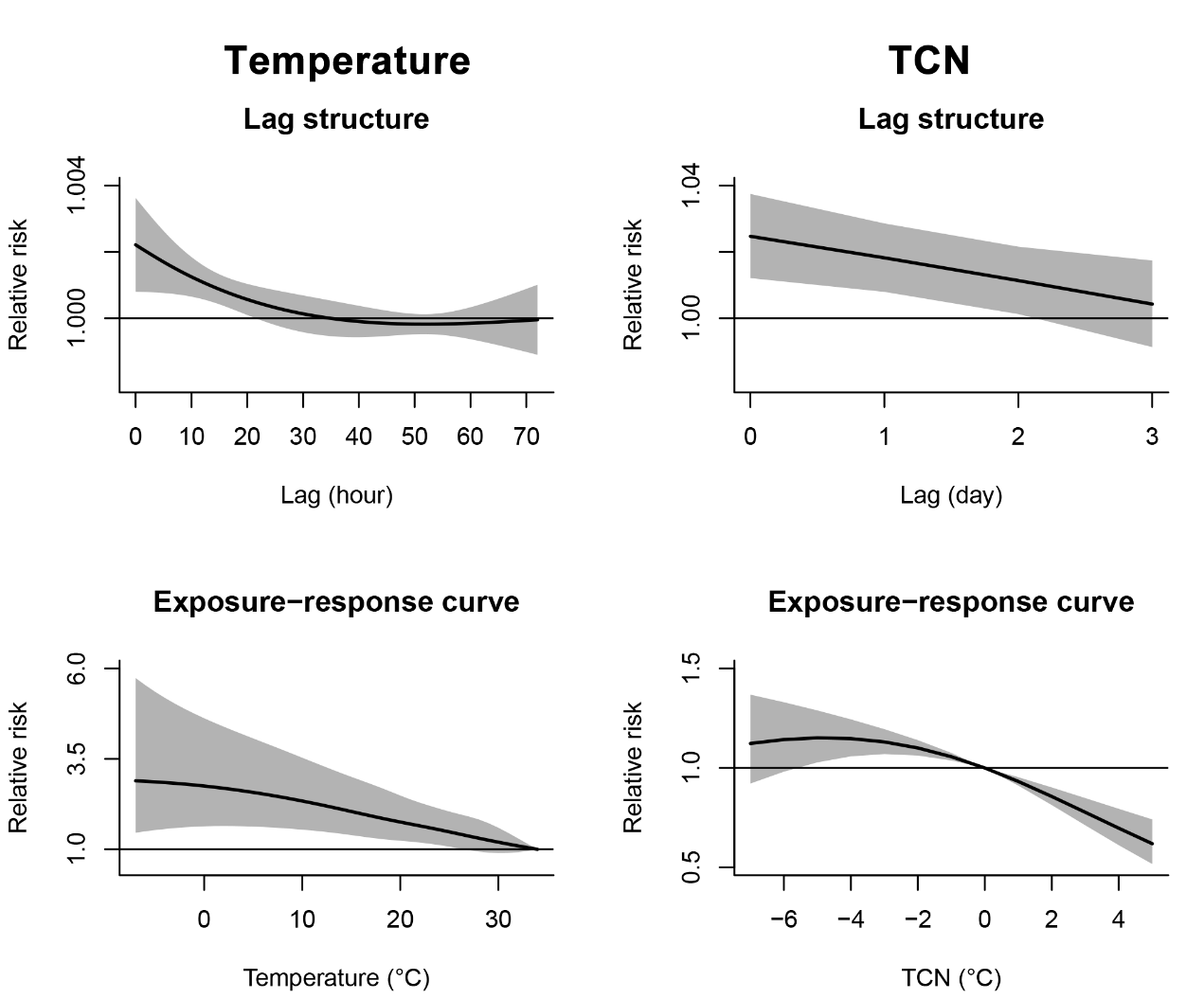


**Online eFig 1. Exposure-response relationships and Lag structure for temperature and TCN with acute aortic dissection in pilot study.** TCN, temperature change between neighboring days; Effects were estimated as relative risk per 1℃ lower temperature or temperature decline between neighboring days in lag structure; Solid lines=relative risk of aortic dissection; shaded areas=95% confidence intervals.

Note: The pilot study included acute aortic dissection cases occurred from January 2015 to September 2020 in hospitals certified by Chinese Cardiovascular Association in three provinces (Hebei, Zhejiang and Guangdong), which are located in the Northern, Central and Southern China respectively. A total of 8,048 eligible cases from 334 hospitals were finally included in analysis.


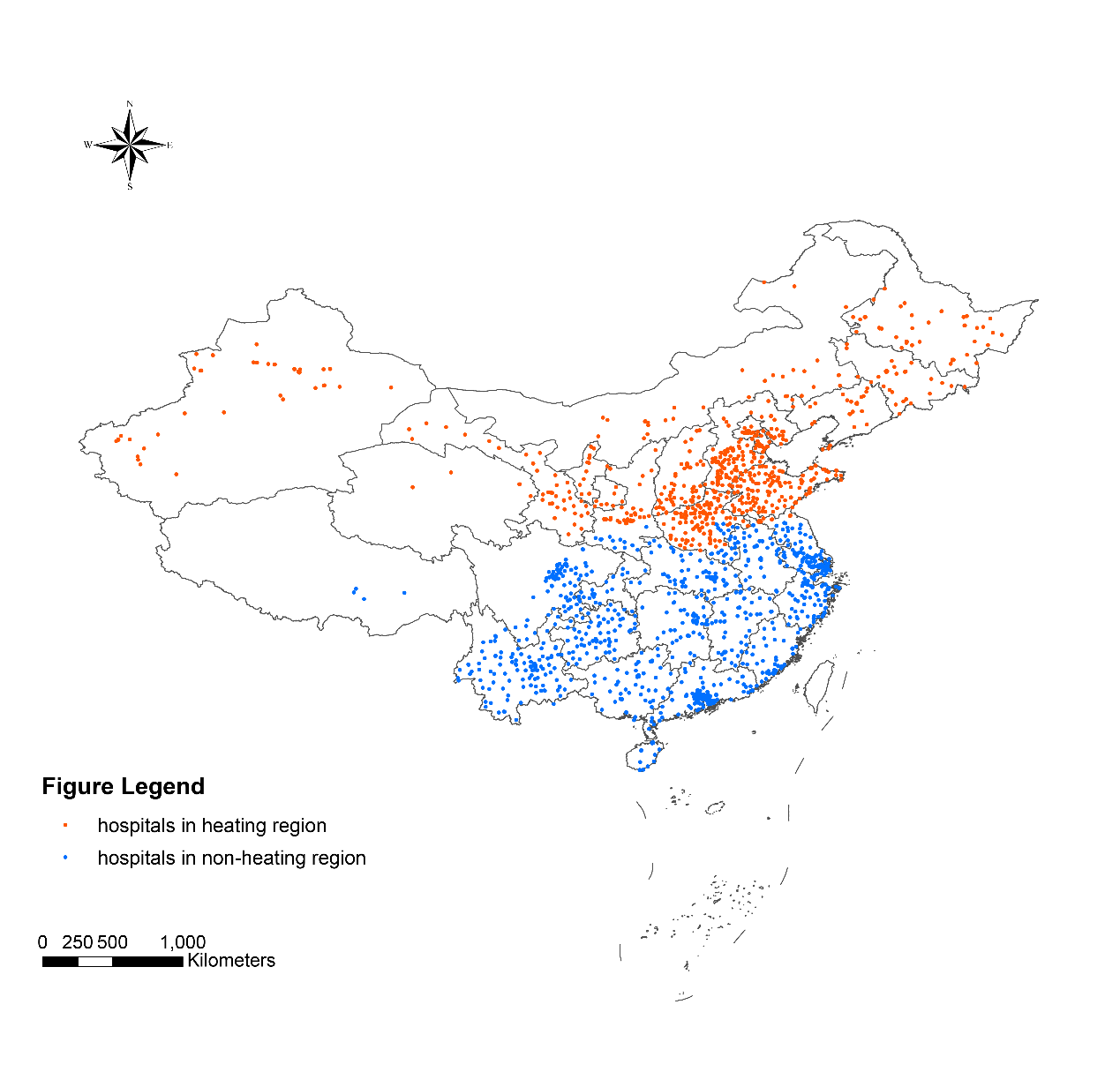


**Online eFig 2. locations of participating hospitals in the CCA database.**

**
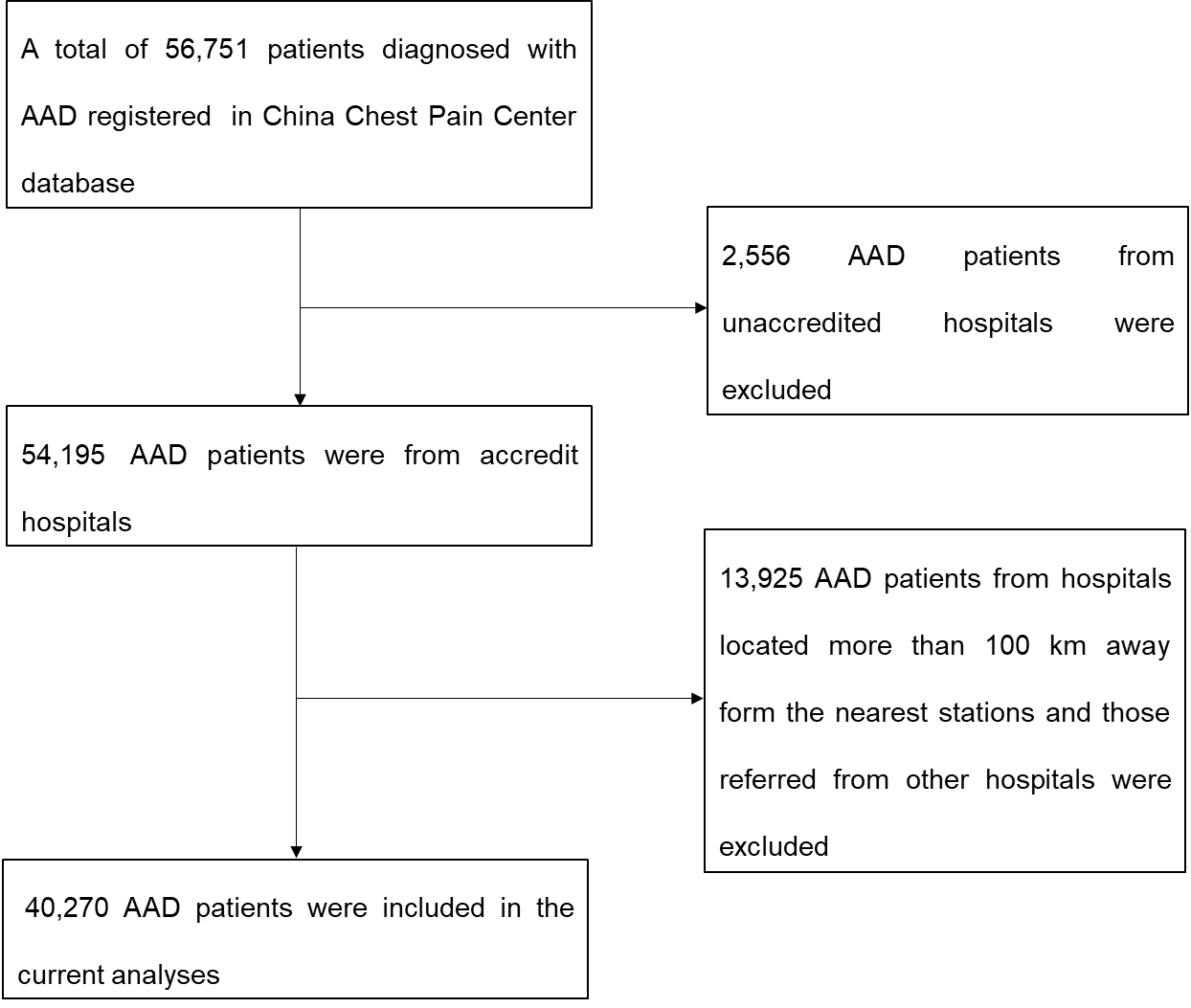
Online eFig 3. Inclusion and exclusion of acute aortic dissection patients from the CCA database**


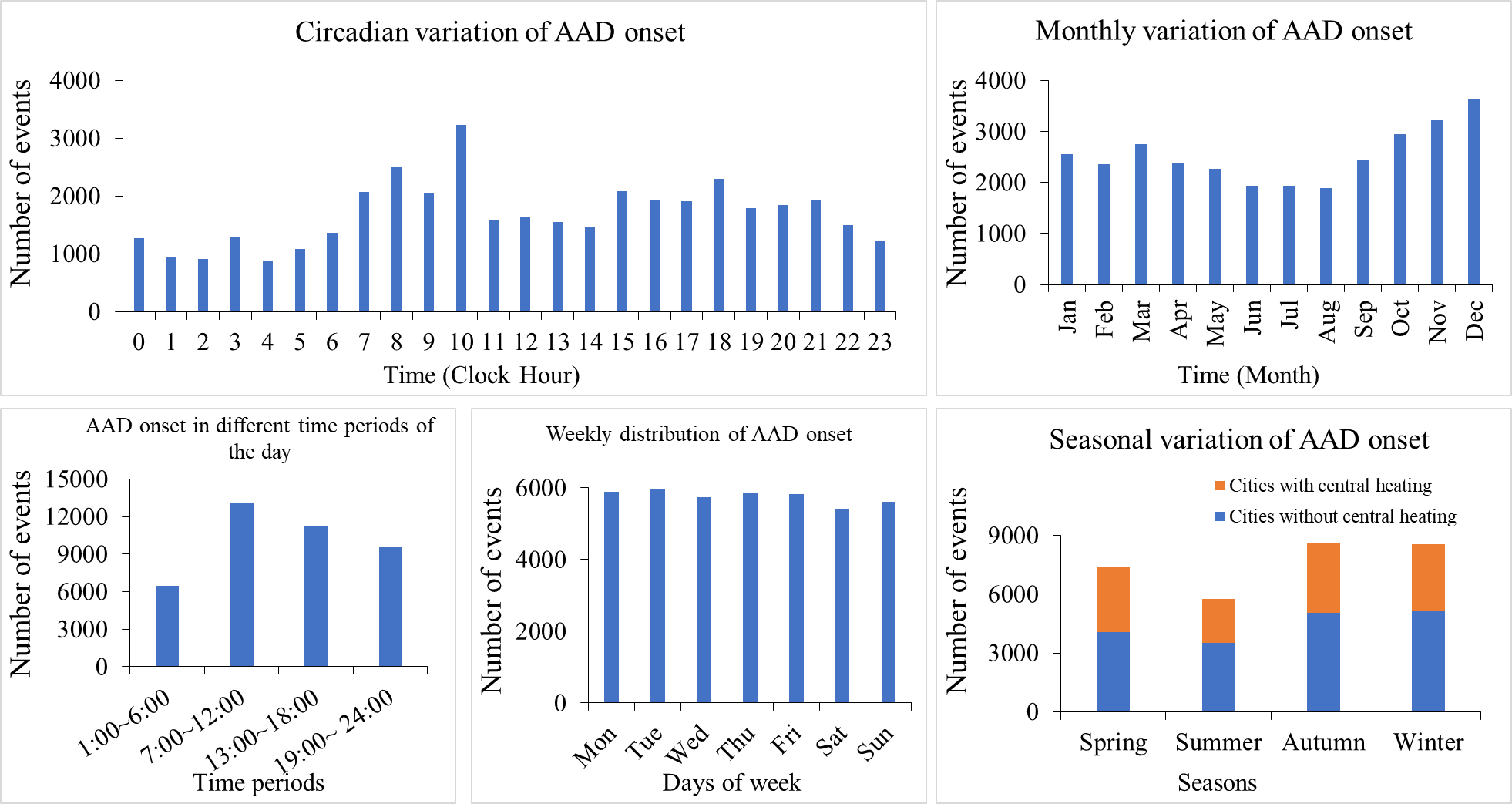


**Online eFig 4. Time distribution of acute aortic dissection onset.** Cases occurred in 2020 were excluded in the description of seasonal and monthly variation, because data for 2020 was not for a whole year, and only cases occurred before September 2020 were available till the start of this analysis.


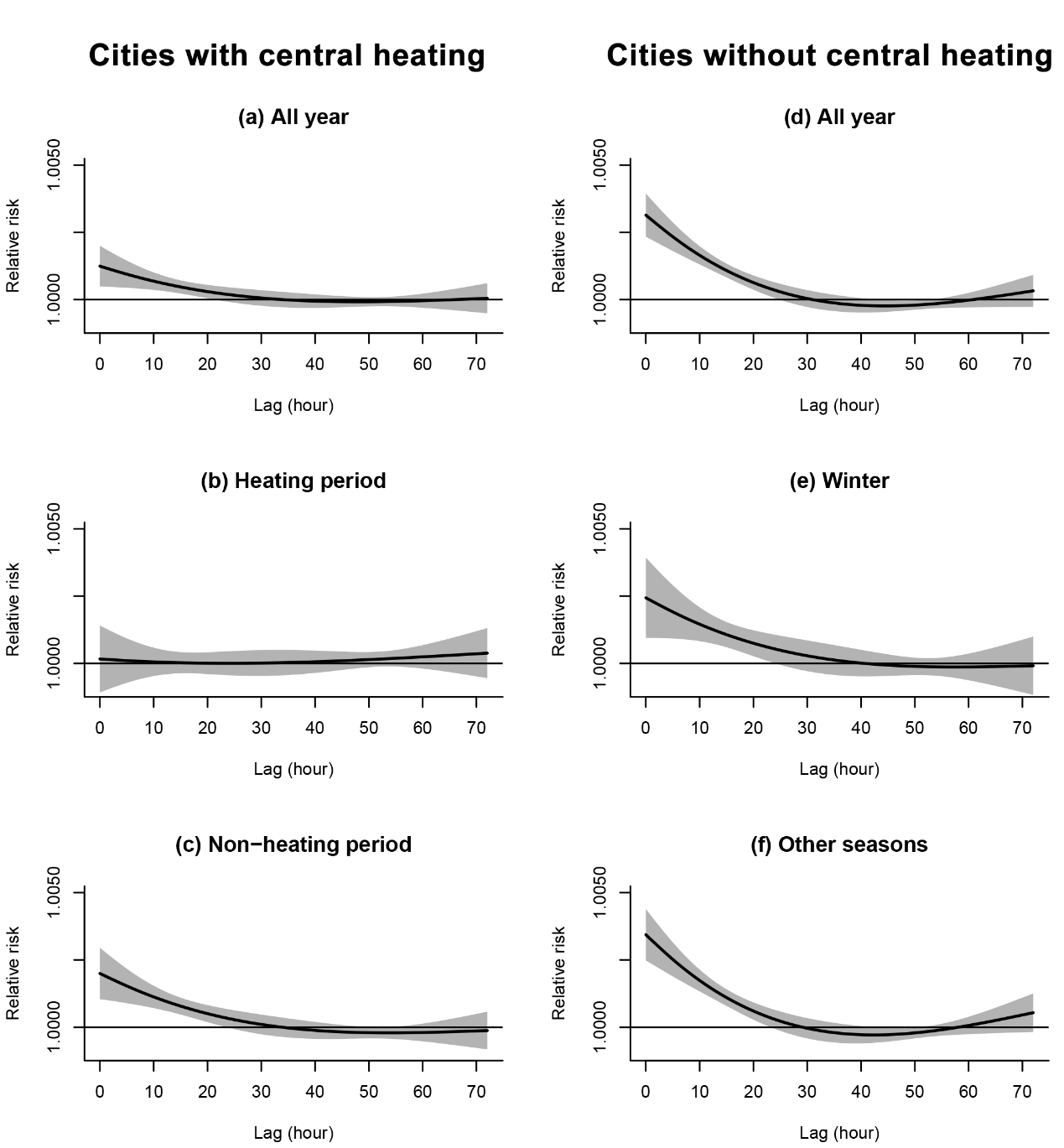


**Online eFig 5. Lag structure for temperature and acute aortic dissection during all year (a), heating periods (b), and non-heating periods (c) in cities with central heating, and during all year (d), winter (e), and other seasons (f) in cities without central heating.** Effects were estimated as relative risk per 1℃ decreases in temperature; Solid lines=relative risk of aortic dissection; shaded areas=95% confidence intervals


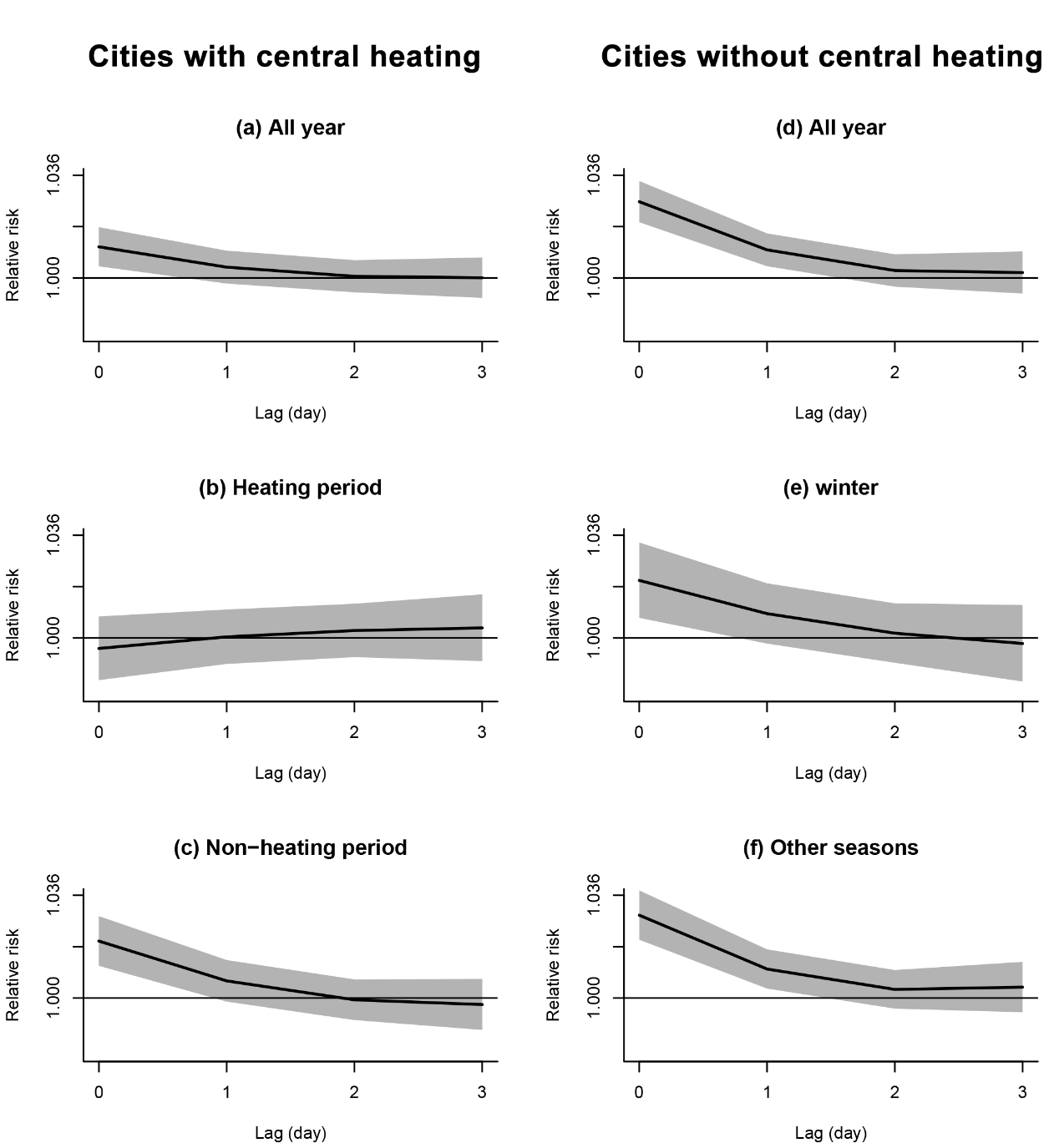


**Online eFig 6. Lag structure for TCN and acute aortic dissection during all year (a), heating periods (b), and non-heating periods (c) in cities with central heating, and during all year (d), winter (e), and other seasons (f) in cities without central heating.** TCN, temperature change between neighboring days; Effects were estimated as relative risk per 1℃ decreases in daily mean temperature from the previous day; Solid lines=relative risk of aortic dissection; shaded areas=95% confidence intervals


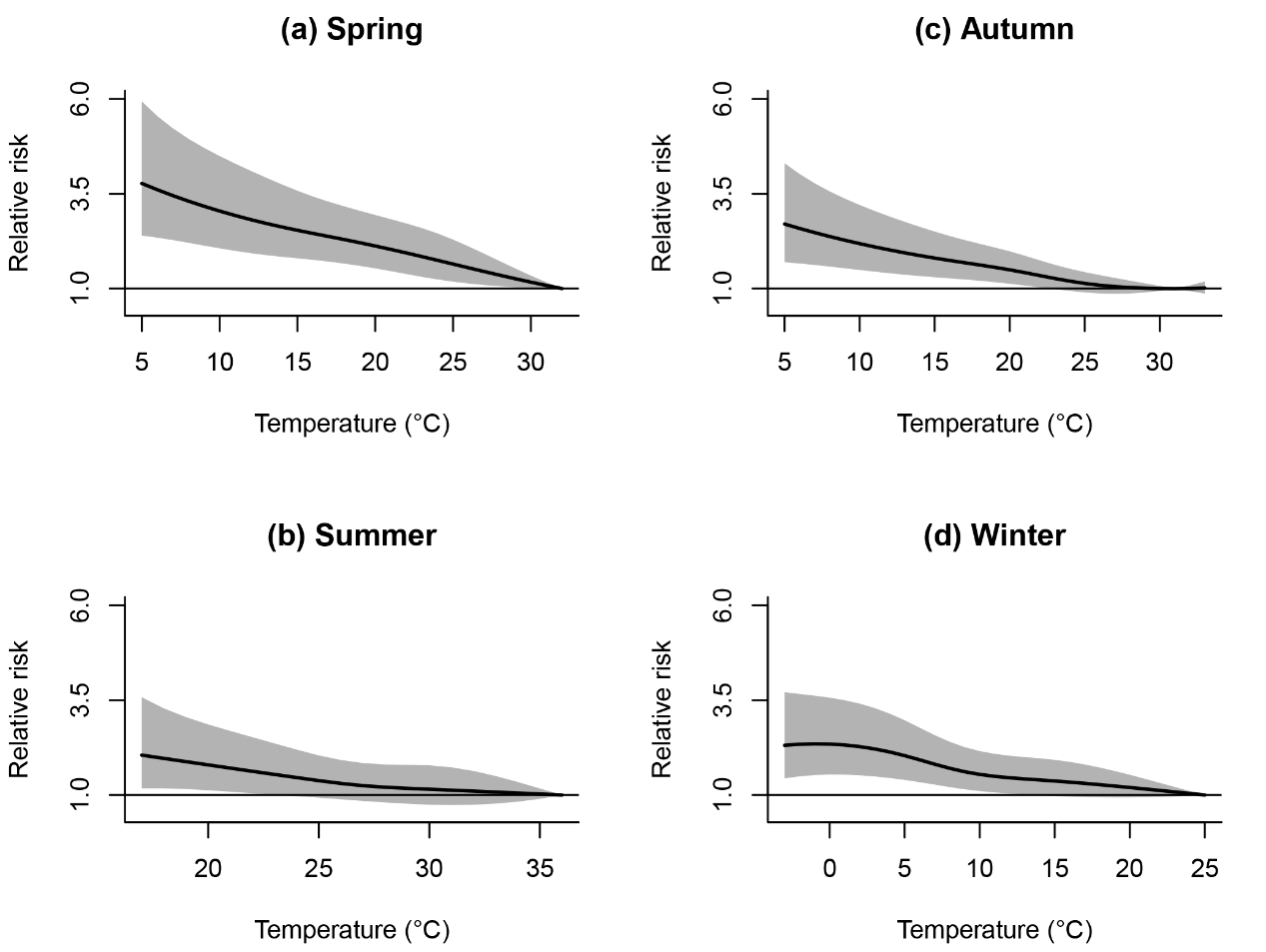


**Online eFig 7. Exposure-response curves for temperature and acute aortic dissection at lag 0-24 hours in spring (a), summer (b), autumn (c), and winter (d).** Solid lines=relative risk of aortic dissection; shaded areas=95% confidence intervals.


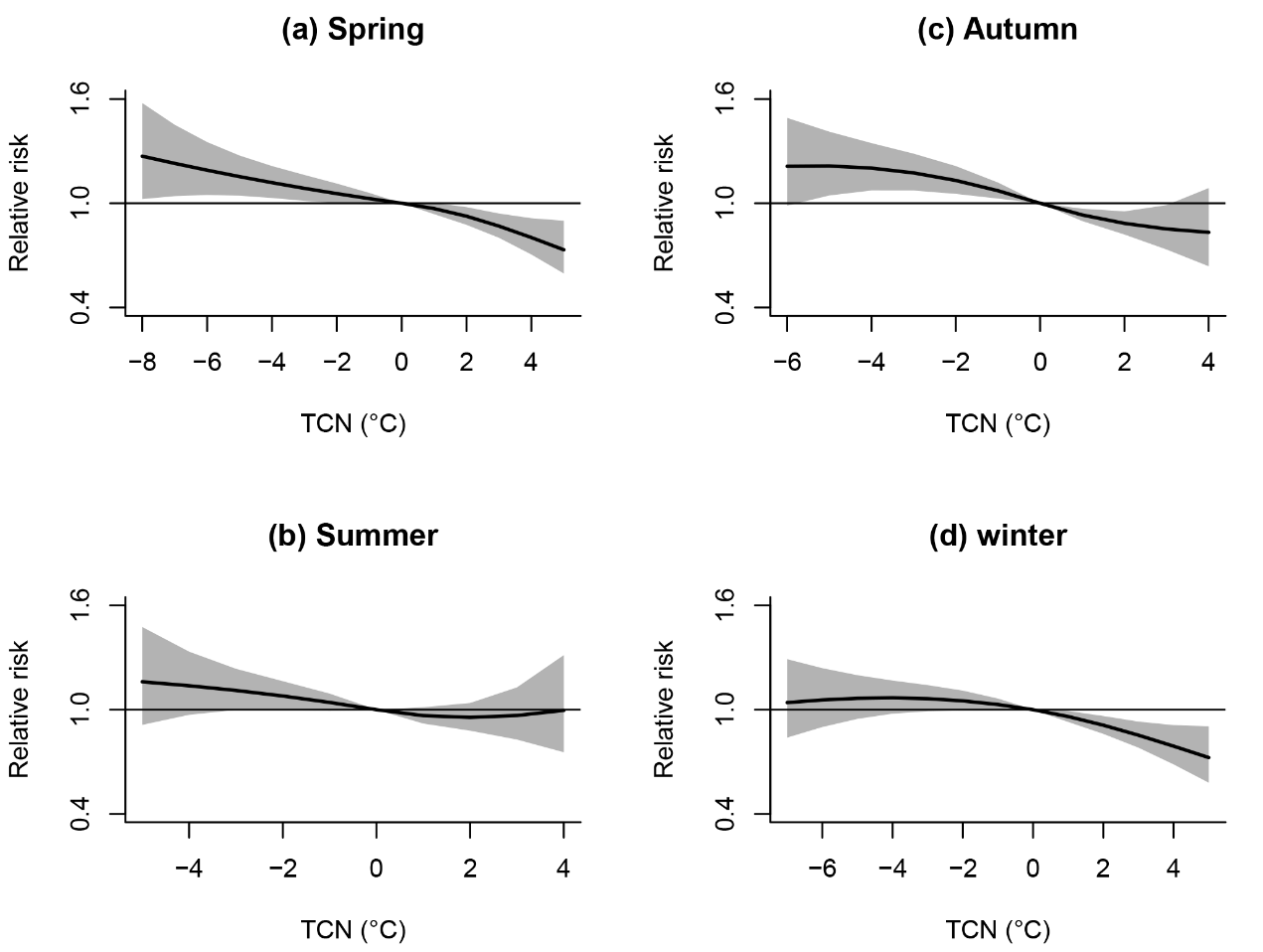


**Online eFig 8. Exposure-response curves for TCN and acute aortic dissection at lag 0-1 days in spring (a), summer (b), autumn (c), and winter (d).** TCN, temperature change in neighboring days. Solid lines=relative risk of aortic dissection; shaded areas=95% confidence intervals.


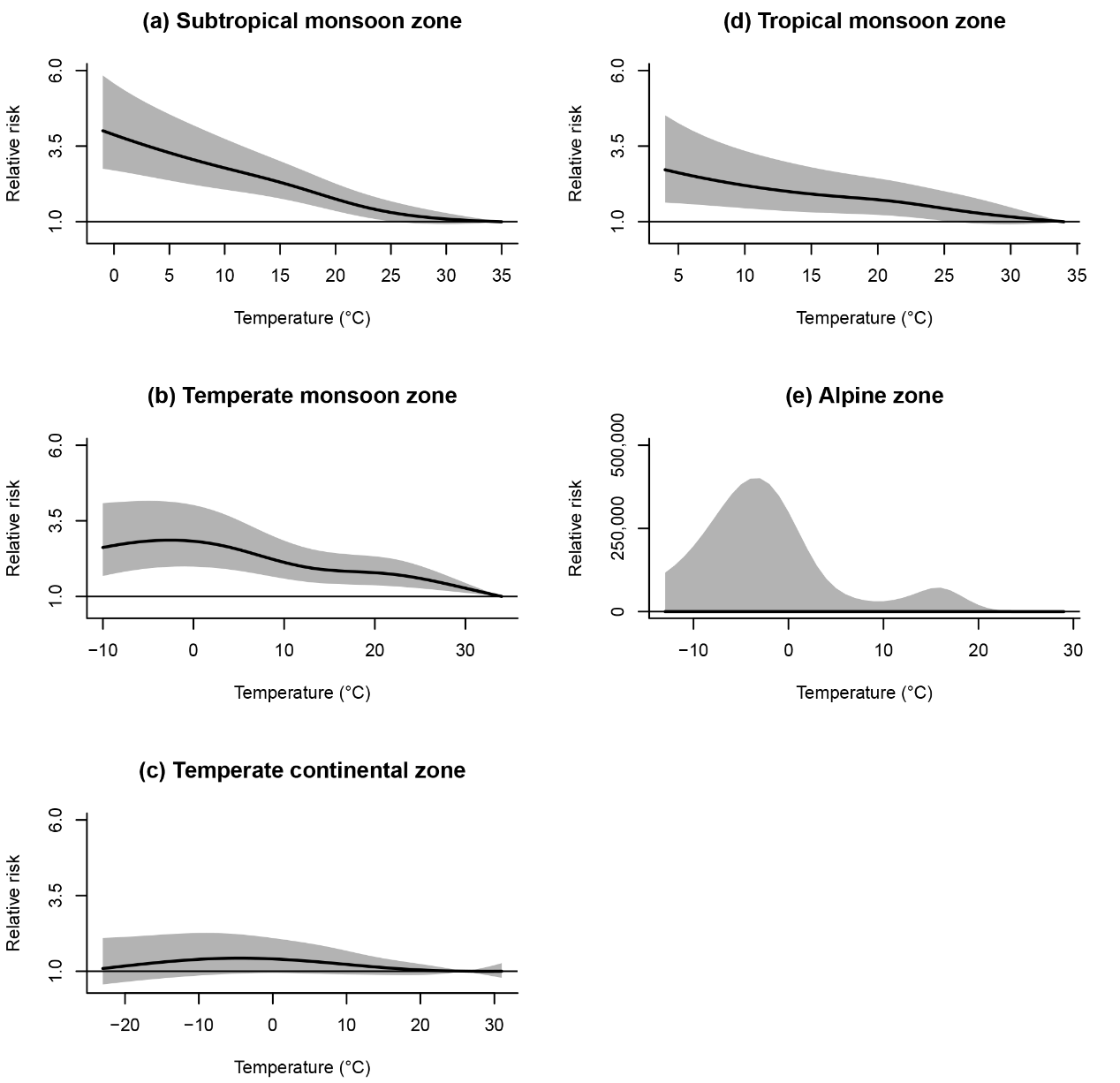


**Online eFig 9. Exposure-response curves for temperature and acute aortic dissection at lag 0-24 hours in subtropical monsoon zone (a), the temperate monsoon zone (b), the temperate continental zone (c), the tropical monsoon zone (d), and alpine zone (e).** Solid lines=relative risk of aortic dissection; shaded areas=95% confidence intervals.


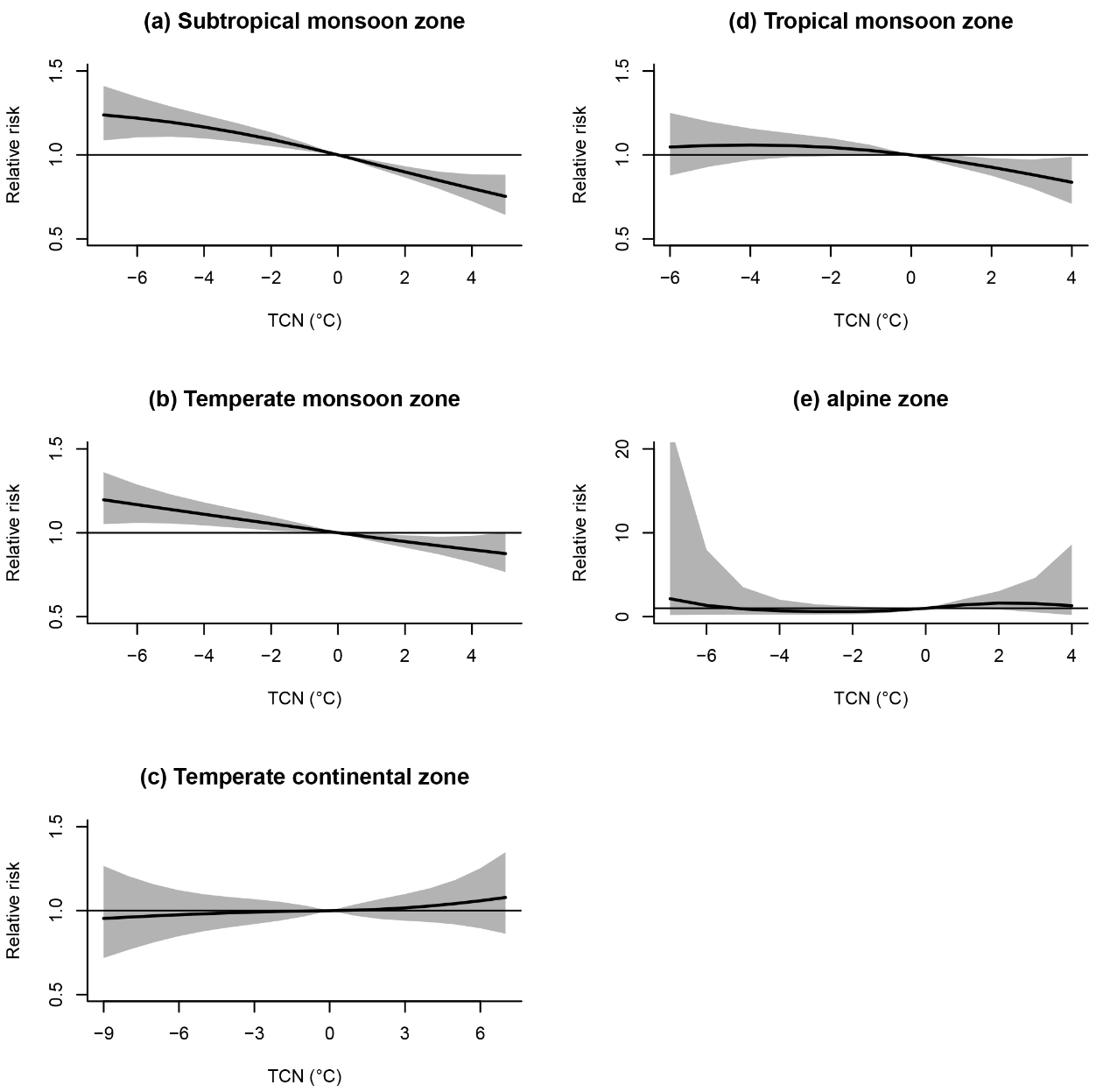


**Online eFig 10. Exposure-response curves for TCN and** **acute aortic dissection at lag 0-1 days in subtropical monsoon zone (a), the temperate monsoon zone (b), the temperate continental zone (c), the tropical monsoon zone (d), and alpine zone (e).** TCN, temperature change in neighboring days. Solid lines=relative risk of aortic dissection; shaded areas=95% confidence intervals.


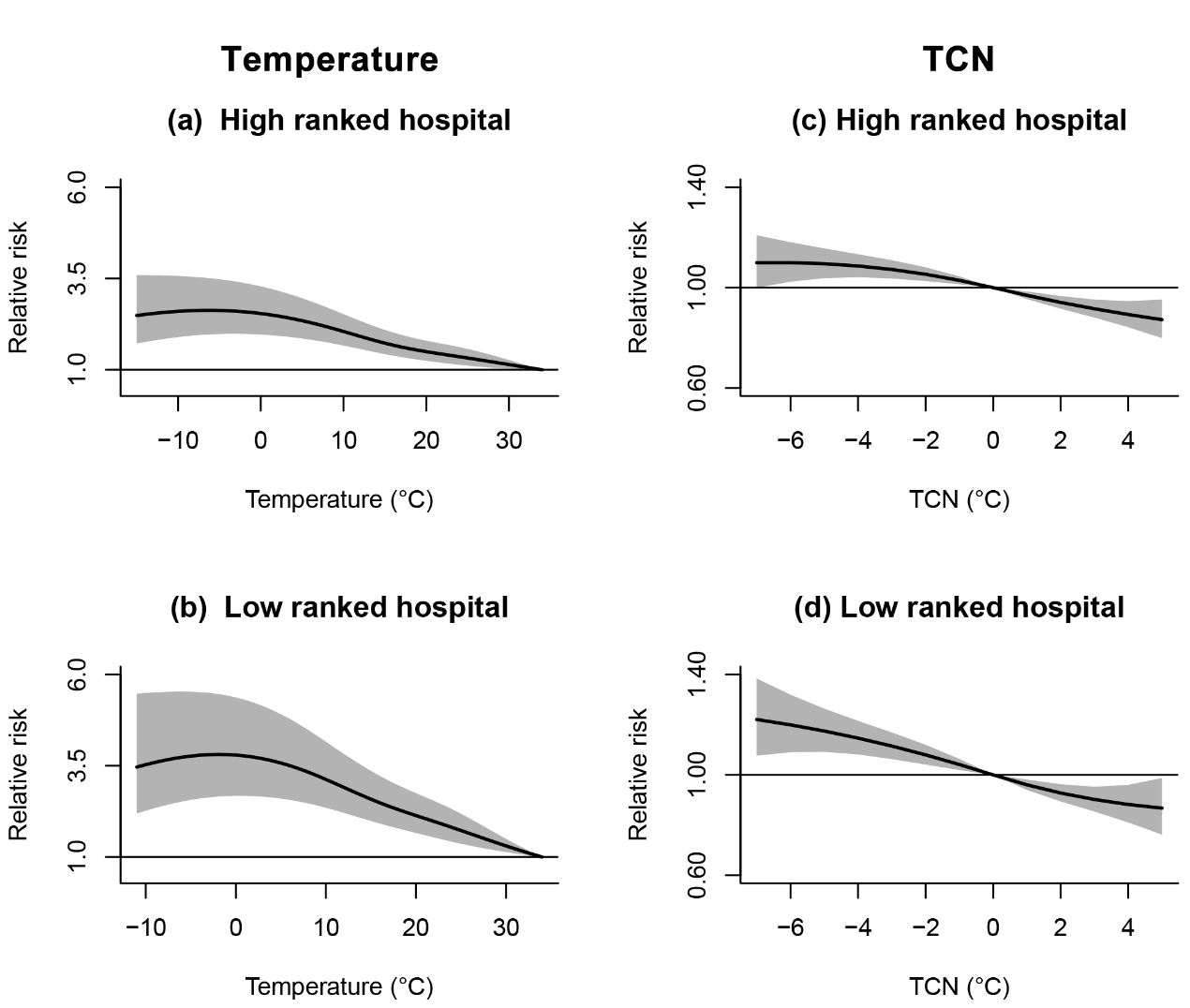


**Online eFig 11. Exposure-response relationships for temperature (lag 0-24 hours) and TCN (lag 0-1 days) with acute aortic dissection in high-ranked and low ranked hospitals.** TCN, temperature change in neighboring days. Solid lines=relative risk of aortic dissection; shaded areas=95% confidence intervals.


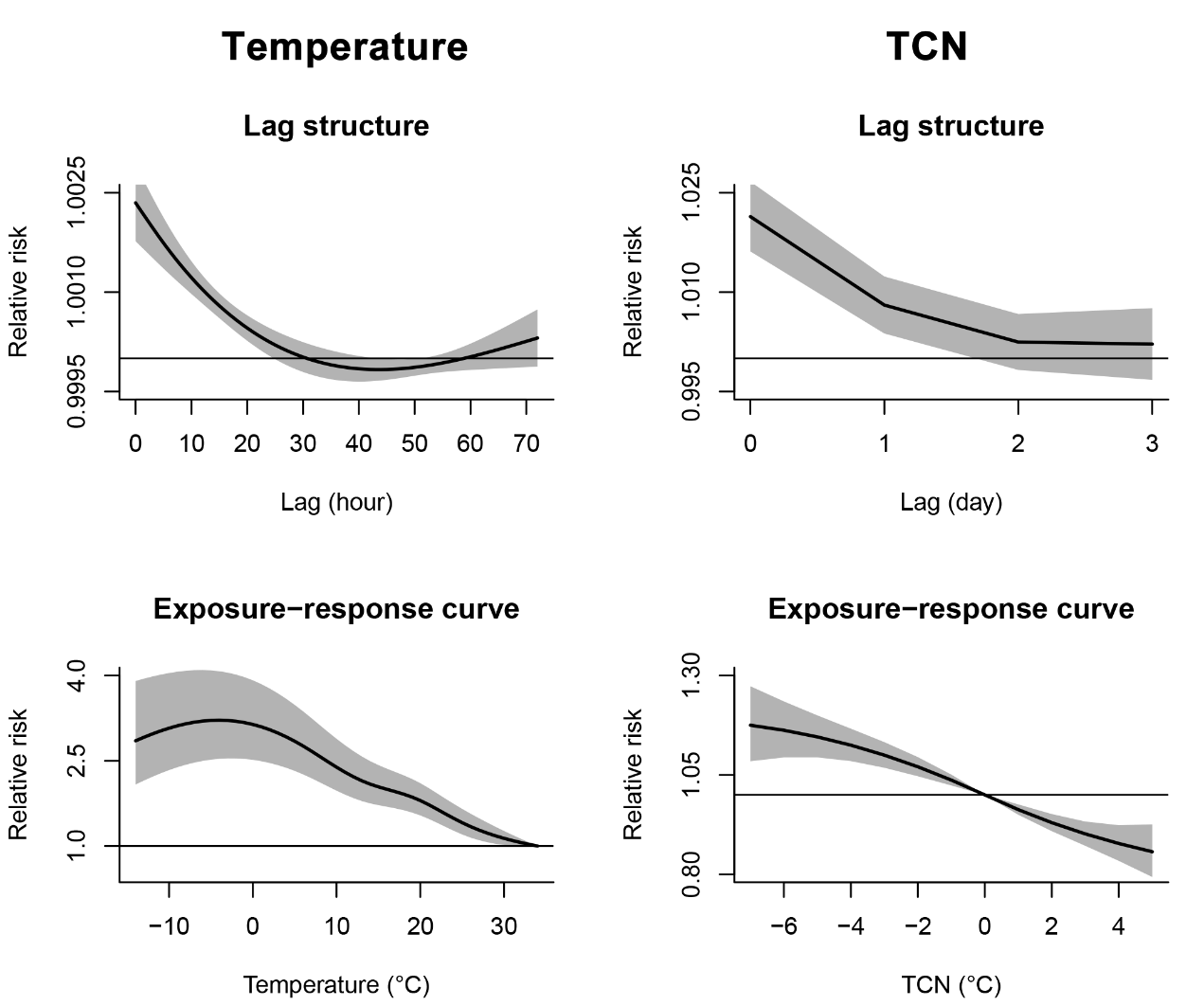


**Online eFig 12. Lag structure and exposure-response relationships for temperature (lag 0-24 hours) and TCN (lag 0-1 days) with acute aortic dissection after excluding cases who admitted to hospital more than 48 hours after initial onset.** Effects were estimated as relative risk per 1℃ lower temperature or temperature decline between neighboring days in lag structure; Solid lines=relative risk of aortic dissection; shaded areas=95% confidence intervals.
